# Functional and Psychosocial Changes Following a Culturally Adapted Folk Dance Program in Community-Dwelling Older Adults

**DOI:** 10.3390/geriatrics11050149

**Published:** 2026-10-20

**Authors:** Yeny Concha-Cisternas, Fernando Muñoz-Sepúlveda, Eduardo Guzmán-Muñoz, Eugenio Merellano-Navarro, Lincoyán Fernández-Huerta, Daniela Robles Tapia, Walter Sepúlveda-Loyola, Felipe Montalva-Valenzuela, Rodrigo Yañez-Sepúlveda, Jessica Ibarra-Mora

**Affiliations:** 1 Escuela de Kinesiología, Facultad de Salud, Universidad Santo Tomás, Talca 3460000, Chile; yenyconchaci@santotomas.cl (Y.C.-C.); eguzmanm@santotomas.cl (E.G.-M.); 2 Vicerrectoría de Investigación e Innovación, Universidad Arturo Prat, Iquique 1100000, Chile; 3 Pedagogía en Educación Física, Facultad de Educación, Universidad Autónoma de Chile, Talca 3460000, Chile; 4 Department of Physical Activity Sciences, Faculty of Education Sciences, Universidad Católica del Maule, Talca 3480112, Chile; emerellano@ucm.cl; 5 Escuela de kinesiología, Facultad de Ciencias de la Rehabilitación y Calidad de Vida, Universidad San Sebastián, Concepción 4080871, Chile; lincoyan.fernandez@uss.cl; 6 Escuela de Nutrición y Dietética, Facultad de Salud, Universidad Santo Tomás, Antofagasta 1240000, Chile; danielaroblesta@santotomas.cl; 7 Faculty of Health and Social Sciences, Center for Research in Biological and Chemical Sciences, Universidad de Las Américas, Santiago 7500975, Chile; wsepulveda@udla.cl; 8 Escuela de Entrenador en Actividad Física y Deporte, Facultad de Ciencias Humanas, Universidad Bernardo O’Higgins, Santiago 8320000, Chile; felipe.montalva@ubo.cl; 9 Facultad de Educación y Ciencias Sociales, Universidad Andrés Bello, Viña del Mar 2200055, Chile; rodrigo.yanez.s@unab.cl; 10 School of Medicine, Universidad Espíritu Santo, Samborondón 092301, Ecuador; 11 Departamento de Educación Física, Deportes y Recreación, Universidad Metropolitana de Ciencias de la Educación, Santiago 7760197, Chile

**Keywords:** aged, exercise therapy, dancing, motor activity, quality of life

## Abstract

**Background:** Population ageing requires community-based strategies supporting functional capacity, psychological well-being, and participation among older adults. **Objective:** To analyze the changes following a community-based motor education program grounded in Chilean folk dances in physical function, psychological well-being, and subjective participation experiences among older adults. **Methods:** A sequential explanatory mixed-methods design included a single-group pre–post quantitative phase and a qualitative phase. Physical function was assessed using the Short Physical Performance Battery (SPPB), Timed Up and Go (TUG), and single-leg stance tests. Psychological outcomes included the SF-12, life satisfaction, self-efficacy, and perceived social support. The 9-week intervention comprised two 90-min sessions weekly. Sixty-four older adults were recruited: 58 completed pre–post assessments and 17 joined a focus group. Quantitative changes were examined using the Wilcoxon signed-rank test, and qualitative data were analyzed thematically. **Results:** Mean age was 72.1 years, and 89.7% were women. Significant improvements were observed in SPPB scores (*p* = 0.003), dominant single-leg stance (*p* < 0.001), non-dominant single-leg stance (*p* < 0.001), SF-12 Mental Component (*p* < 0.001), self-efficacy (*p* = 0.010), and perceived social support (*p* < 0.001). No significant changes were found in TUG, the SF-12 Physical Component, or life satisfaction. Qualitative findings emphasized the instructor’s role, reduced embarrassment and fear of ridicule, and stronger social bonds. **Conclusions**: Participation was associated with improvements in physical and psychological outcomes, supporting culturally adapted folk dance-based motor education as a feasible strategy for active and healthy ageing.

## 1. Introduction

Population ageing represents one of the most significant demographic transformations of the 21st century. It is estimated that by 2050, one in six people worldwide will be over 65 years of age, doubling the proportion recorded in 2019 [1], a phenomenon largely attributable to advances in health care, access to basic services, and improvements in the social determinants of health [2]. This demographic transition not only increases longevity but also challenges health systems and communities to promote physical function, autonomy, and sustained participation in later life. In parallel, a shift has occurred in the conceptualization of ageing, moving away from deficit-oriented perspectives toward approaches centered on capacities, autonomy, and the active participation of older adults in society [3].

Within this framework, the World Health Organization (WHO) has promoted the active ageing paradigm, defined as the process of optimizing opportunities for health, participation, and security to enhance quality of life in older age [4]. This approach recognizes the value of lifelong learning to maintain functionality, preserve mental health, and foster social integration [5,6]. These dimensions are closely linked to psychological well-being in later life, understood as a multidimensional experience involving how individuals feel and evaluate their lives, including life satisfaction, emotional well-being, and a sense of meaning or purpose [7,8]. Educational participation may contribute to these dimensions by providing opportunities for personal development, social interaction, and continued engagement in meaningful activities. In this context, participation in educational programs, both formal and non-formal, has been shown to have a positive impact on older adults, as evidenced by improvements in psychosocial outcomes such as life satisfaction, self-efficacy, perceived social support, and sense of purpose [9,10,11,12]. However, access to such opportunities remains uneven across settings and populations.

In Chile, access of older adults to educational opportunities remains limited, as although 41% report interest in learning new topics, only 15% participate, primarily in areas such as computer skills, crafts, health, and movement [13]. This gap is particularly relevant in a country with a high prevalence of multimorbidity, functional decline, and mental health problems in older age [14,15], reinforcing the need to expand and diversify the educational offer directed at this age group. Addressing this gap is therefore both a public health priority and a matter of social equity.

Among emerging alternatives in the field of education for older adults, motor education courses have gained relevance due to their capacity to promote meaningful learning through movement, in close connection with culture and collective life [16]. These courses are grounded in the embodied learning approach, which recognizes the body as the central axis of the educational experience and as a vehicle for integrating motor, cognitive, emotional, and social dimensions [17]. From this perspective, knowledge is not transmitted verbally or abstractly, but rather constructed through situated, culturally relevant, and shared bodily experiences, thereby fostering motor development, cognitive stimulation, social interaction, and a sense of belonging—dimensions that are fundamental for preserving autonomy and well-being in older age [17,18]. In contrast to interventions focused exclusively on physical performance, motor education proposes pedagogical spaces in which the moving body facilitates the construction of shared meanings, emotional expression, and active participation in everyday and symbolic contexts [19]. This perspective is also consistent with evidence from neuroscience showing that dance constitutes a cognitively enriched motor-learning activity, as learning and adapting movement sequences simultaneously involves memory, rhythmic processing, spatial orientation, coordination, and sensorimotor integration. In older adults, dance programs involving novel and progressively challenging choreographies have been associated with structural and functional neuroplastic changes [20,21].

A culturally grounded and promising pathway for implementing this approach in programs for older adults is the incorporation of traditional folk dance, which combines structured motor practice with culturally familiar music and movement patterns and simultaneously challenges balance, coordination, mobility, and lower-limb control while providing opportunities for social interaction and emotional expression. These characteristics provide a plausible pathway through which dance may influence both physical function and psychosocial well-being. Evidence has shown benefits for mobility, balance, and quality of life [22,23]. This pedagogical resource not only allows the integration of movement with emotion, memory, and identity, but also provides a rhythmic and narrative structure that enriches the embodied learning experience [5]. By including folklore within programs of intentional movement, motor, cognitive, and affective dimensions are simultaneously strengthened, while cultural identity and group cohesion are reinforced [24]. Evidence indicates that interventions incorporating traditional dances and popular motor repertoires significantly improve variables such as balance, muscular strength, and functional mobility, with effect sizes comparable to or even greater than those observed in other forms of physical activity [25,26,27,28]. These benefits are not restricted to the physical domain, but also extend to psychological well-being, perceived social support, and motivation for practice. Recent evidence syntheses further support this multidimensional potential of dance in older adults, reporting benefits across physical function, balance, quality of life, and psychosocial well-being [27,29,30]. Consistent findings have also been reported in culturally specific interventions, including Turkish and Greek traditional dance programs, with improvements in physical performance, balance, physical fitness, and well-being [18,25].

Therefore, integrating community-based motor education with folkloric expressions not only addresses a functional need, but also represents an opportunity to promote active ageing. Considering the available evidence, it is a priority to advance in the development and evaluation of strategies that articulate body, culture, and community as central axes of well-being in older age, particularly in contexts such as Chile, where educational gaps and health conditions among older adults make a more comprehensive approach urgently needed. Beyond evaluating dance solely as a form of physical activity, the present study examines a culturally adapted motor-education model that integrates structured movement practice, folk-dance learning, theoretical–reflective content, and collective participation. Its contribution lies in linking objectively measured functional and psychosocial changes with participants’ accounts of technical learning, emotional change, cultural meaning, and social connection within the same explanatory mixed-methods framework. Accordingly, the objective of this study is to analyze the changes following a community-based motor education program grounded in Chilean folk dances in physical function, psychological well-being, and subjective participation experiences among older adults.

## 2. Materials and Methods

### 2.1. Study Design and Setting

This study was conducted using a mixed-methods sequential explanatory design, comprising a single-group pre–post quasi-experimental quantitative phase followed by a complementary qualitative phase. The qualitative phase adopted an interpretative approach aimed at understanding participants’ perceptions, meanings, and subjective experiences following the folk-dance intervention. A focus group was conducted at the end of the program to collect collective discourses regarding technical learning, emotional impact, and pedagogical appraisal of the workshop. The interpretative approach was grounded in the search for an in-depth understanding of lived experiences from the participants’ own perspectives [28], while the use of focus groups was justified by their capacity to promote discursive interaction and reveal shared meanings in educational contexts [31,32].

### 2.2. Participants and Outcomes

The target population consisted of older adults registered in community centers in a city in central Chile. Participants were community-dwelling older adults attending the participating centers, which constituted the recruitment setting for the study. Enrollment was open to eligible adults aged ≥ 60 years, regardless of sex, who met the functional and cognitive criteria described below.

For the quantitative phase, sample size was estimated using G*Power 3.1 software (University of Düsseldorf, Düsseldorf, Germany), based on the five-repetition chair-rise test reported by Eyigor et al. [17]. In that randomized trial, post-intervention chair-rise time was 8.3 ± 1.0 s in the folk-dance group and 10.7 ± 2.5 s in the control group, corresponding to a between-group difference of 2.4 s. The chair-rise test was selected because it provides an objective measure of lower-extremity functional performance and is directly related to the chair-rise component of the SPPB used in the present study. A pooled standard deviation of approximately 1.88 s, a two-tailed significance level of 0.05, and a statistical power of 90% were considered. The calculation indicated a minimum requirement of 13 participants; after allowing for an estimated attrition rate of 15%, the minimum recruitment target was increased to 16 participants. This value was treated as the minimum required sample rather than a recruitment ceiling. Recruitment therefore continued among eligible older adults available through the participating community centers, increasing the precision of the estimates and providing additional protection against losses to follow-up. Consequently, 64 participants were enrolled.

For the qualitative phase, all participants who expressed willingness to take part in a post-intervention focus group were included in order to explore their perceptions and subjective experiences. A total of 17 older adults participated in the focus group, which allowed the collection of collective discourses regarding technical learning, emotional impact, and pedagogical appraisal of folk dances as an educational and recreational resource.

Inclusion criteria were: (a) age ≥ 60 years, regardless of sex; (b) residence in the study area; (c) functional condition compatible with physical activity practice, determined using the Functional Examination of Older Adults (EFAM-Chile) [33]; and (d) absence of suspected major or minor neurocognitive disorder, assessed using the Abbreviated Mini-Mental State Examination [34]. Exclusion criteria included: (a) self-reported uncontrolled medical conditions contraindicating physical activity (e.g., unstable cardiac disease, severe respiratory insufficiency, or other limiting conditions); and (b) active participation in other physical intervention programs with similar characteristics during the study period.

The study was conducted in accordance with the principles of the Declaration of Helsinki. All participants were informed about the objectives, procedures, benefits, and potential risks of the study and provided written informed consent. The study protocol was approved by an institutional scientific ethics committee (Approval Nº. CEC 39-24).

Quantitative Phase

### 2.3. Outcome and Measures

Physical function and psychological well-being were assessed using standardized instruments, including the Short Physical Performance Battery (SPPB), the Timed Up and Go (TUG) test, and the single-leg stance test.

#### 2.3.1. Short Physical Performance Battery (SPPB)

This instrument assesses functional physical performance through three tests: static balance (feet together, semi-tandem, and tandem positions), habitual gait speed over a 4-m distance, and repeated chair stands performed five times without using the arms [35]. Each test is scored from 0 to 4 points according to normative criteria, and the total score is obtained by summing the three components (range: 0–12 points), with higher values indicating better physical performance [36].

#### 2.3.2. Timed Up and Go (TUG)

The Timed Up and Go (TUG) test is a clinical tool used to assess functional mobility, dynamic balance, and fall risk in older adults [37,38]. It measures the time, in seconds, required for an individual to rise from a chair without armrests, walk 3 m, turn, return to the starting point, and sit down again. Timing begins when the participant’s back leaves the chair backrest and ends when the initial seated position is resumed.

#### 2.3.3. Single-Leg Stance Test

This test assesses static balance [37]. Participants stood with arms crossed over the chest and hands resting on the shoulders, maintaining their gaze forward. They were instructed to raise one leg to approximately 90° of hip and knee flexion and maintain the position as long as possible without external support. Both lower limbs (dominant and non-dominant) were assessed. Time was recorded from foot lift-off until premature contact with the ground or evident loss of balance. The procedure was repeated three times per side, and the mean value was used for analysis [39].

#### 2.3.4. Health-Related Quality of Life

Health-related quality of life was assessed using the Short Form-12 Health Survey (SF-12), a 12-item instrument designed to estimate two main domains: the Physical Component Summary and the Mental Component Summary, reflecting perceived physical and mental health status, respectively [40]. Higher scores indicate better perceived health. The questionnaire includes items addressing physical limitations, emotional functioning, vitality, and mental health; one of the mental-health items asks how frequently the respondent felt calm and peaceful during the previous four weeks. The SF-12 has demonstrated adequate psychometric properties and has been validated in both Hispanic and Chilean populations [41,42].

#### 2.3.5. General Perceived Self-Efficacy

General perceived self-efficacy was assessed using the 10-item General Self-Efficacy Scale developed by Schwarzer and Jerusalem [43]. The Spanish wording of the items was based on the Chilean adaptation reported by Clavijo et al. [44]. The items assess perceived competence in coping with difficult or unexpected situations. A representative item evaluates whether the respondent believes that difficult problems can be solved when sufficient effort is made. In the present study, each item was rated on a 7-point Likert scale ranging from 1 (completely disagree) to 7 (completely agree), yielding a total score from 10 to 70, with higher scores indicating greater perceived general self-efficacy. The response format used in this study differed from the 4-point format employed in the Chilean validation.

#### 2.3.6. Perceived Social Support

Perceived social support was assessed using the Multidimensional Scale of Perceived Social Support, composed of 12 items evaluating support from family, friends, and significant others [45,46]. The items assess the perceived availability of support from these three sources; one item examines whether respondents perceive that their family tries to provide them with help. Items are rated on a 7-point Likert scale, yielding a total score ranging from 12 to 84, with higher scores indicating greater perceived social support. The scale has been validated in Chilean older adults and shows high reliability [47].

#### 2.3.7. Life Satisfaction

Life satisfaction was assessed using the Satisfaction with Life Scale developed by Diener et al. [48] and adapted to Spanish by Pons et al. [49]. The scale consists of five items rated on a 5-point Likert scale, focused on the global cognitive evaluation of one’s life; one item examines whether, in most respects, respondents perceive their life as being close to how they would like it to be. Higher scores indicate greater life satisfaction. Higher scores indicate greater life satisfaction, whereas lower scores reflect lower perceived satisfaction with life.

Qualitative Phase

#### 2.3.8. Focus Group Procedures and Analysis

The qualitative phase followed an interpretive approach to understand participants’ perceptions, meanings, and subjective experiences after participating in the folklore classes. A face-to-face focus group of approximately 50 min was conducted, audio-recorded, and transcribed verbatim for analysis [31]. The focus group guide was developed by the research team and validated through consultation with two doctoral-level experts in qualitative research to ensure relevance, clarity, and sensitivity of the questions [32]. The guide addressed four thematic domains: (1) overall experience in the classes, (2) perceived physical impact, (3) emotional and social impact, and (4) satisfaction and suggestions. Questions were open-ended to facilitate free and reflective expression and were preceded by an introduction explaining the purpose of the session and acknowledging participants’ willingness to contribute.

The focus group was conducted after completion of the intervention, in an environment designed to promote trust and respect, facilitating interaction and the co-construction of shared meanings. Data were analyzed using thematic analysis following Braun and Clarke [50], including open coding, identification of discursive patterns, and development of emergent categories. Two researchers with previous experience in qualitative research independently coded the transcript and developed an initial coding framework. Coding discrepancies were reviewed through iterative discussion, with final decisions reached by consensus; when necessary, the researchers returned to the original transcript to preserve the contextual meaning of participants’ accounts. Both coders were trained in qualitative methods and had no prior relationship with the participants beyond the research context; a reflexive journal was kept throughout the analysis to document interpretive decisions and to make explicit the influence of the researchers’ professional background on coding. This approach was complemented by principles of interpretive phenomenology applied to focus group data [28], enabling a deeper interpretation of subjective experiences and the meanings attributed by participants.

### 2.4. Intervention

**Figure 1:**
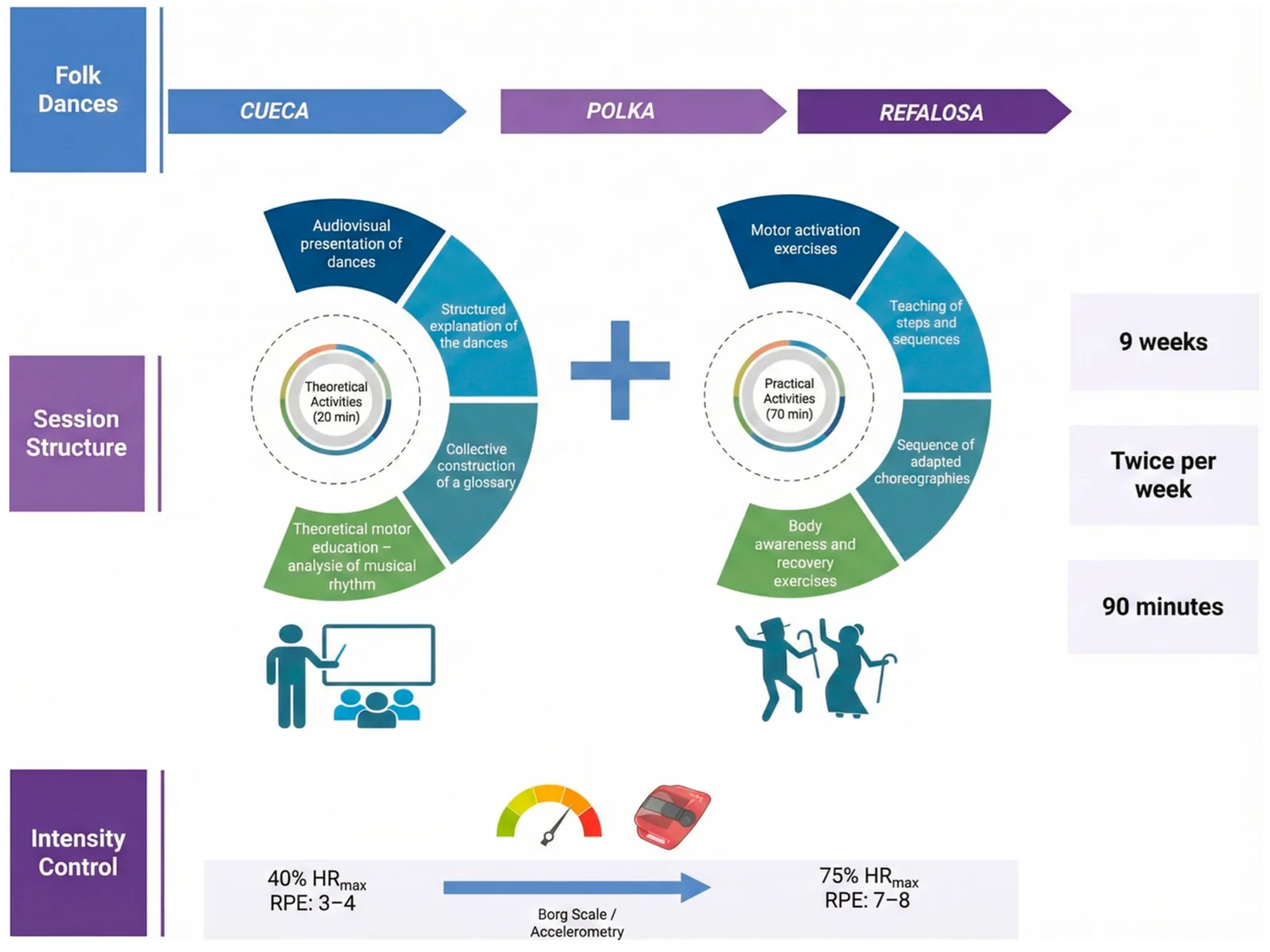
Structure and progression of the culturally adapted folk dance-based motor education program.

The intervention consisted of a theoretical–practical motor education program implemented over 9 weeks, with a frequency of two 90-min sessions per week (18 sessions in total). Each session integrated two complementary components: theoretical activities (20 min) and practical activities (70 min), progressively structured and adapted to participants’ functional characteristics (Figure 1). Beyond motor practice, the program incorporated opportunities for social interaction, shared learning, and collective reflection through its group-based theoretical and practical activities. These components were intended to integrate the physical, educational, and psychosocial dimensions of the intervention throughout the learning process.

Theoretical Activities: During the first 20 min of each session, theoretical activities provided a cultural, technical, and conceptual framework. These included audiovisual presentations of Chilean folk dances (cueca, polka, and refalosa), structured explanations of choreographic structure, musical meter, movement sequences, and basic steps, collective construction of a technical glossary, and motor education content related to musical rhythm, body perception, and spatial orientation. The collective format also provided opportunities to ask questions, discuss the material presented, and share interpretations of the technical and cultural elements addressed during each session.

Practical Activities: Sessions were led by a physical education professional trained in physical activity prescription and folk-dance instruction for older adults and were structured in three phases:

- Activation phase (20 min): Joint mobility, gait, general dynamic coordination, rhythmic displacements, and progressive introduction of basic dance movements.
- Training phase (40 min): Structured learning of the three Chilean folk dances through progressive didactic strategies, including the teaching of basic steps, movement sequences, and adapted choreographies according to participants’ functional capacities. The practical component progressed from basic movements to increasingly complex sequences, with the difficulty of the tasks and choreographies adapted to participants’ functional characteristics. The use of paired and collective dance activities also provided repeated opportunities for interaction and cooperative learning among participants.

Exercise intensity was progressively prescribed and monitored throughout the program. Age-predicted maximal heart rate (HRmax) was estimated using the Tanaka equation (HRmax = 208 − 0.7 × age). Based on this estimation, the target intensity progressed from approximately 40% of HRmax in the initial sessions to 75% of HRmax in the later sessions. Perceived exertion was monitored using the modified Borg CR10 scale, ranging from 3–4 during the initial stages to 7–8 in the final stages of the program. Thus, the intervention followed a progressive intensity pattern from light-to-moderate toward moderate-to-vigorous effort across the 9-week period. Triaxial accelerometers (ActiGraph GT3X+, ActiGraph LLC, Pensacola, FL, USA) were used during selected sessions as a complementary monitoring tool to support real-time adjustments in task complexity and movement demands. Accelerometry data were not considered study outcomes and were not included in the inferential analyses.

- Recovery phase (10 min): Breathing exercises, stretching, and collective reflection on learning and experiences. This reflective period allowed participants to discuss perceived difficulties, achievements, and experiences arising during the sessions, providing a shared space to integrate the motor, emotional, and social aspects of the learning process.

Adherence was recorded through attendance (minimum 75% of sessions). Attendance was registered for each session, and adherence was defined a priori as participation in at least 75% of the 18 scheduled sessions (≥14 sessions). To support continued participation, task complexity, choreographic demands, and exercise intensity were progressively adapted to participants’ functional characteristics throughout the program. At the end of the 9-week program, all pre-established assessments were repeated under standardized conditions to evaluate changes in physical function and psychological well-being.

### 2.5. Data Analysis

Quantitative data were analyzed using GraphPad Prism software, version 9.0 (GraphPad Software, San Diego, CA, USA). The quantitative analysis was restricted to the 58 participants who completed both pre- and post-intervention assessments; no missing outcome data were imputed. Sample characteristics were described using means and standard deviations. Data normality was assessed using the Kolmogorov–Smirnov test. As the data did not follow a normal distribution, the Wilcoxon signed-rank test was used to compare pre- and post-intervention measurements. Paired mean differences with 95% confidence intervals were presented in estimation plots. Effect sizes were calculated as Cohen’s d, defined as the mean pre–post difference divided by the square root of the average of the pre- and post-intervention variances; values of 0.20, 0.50, and 0.80 were interpreted as small, moderate, and large, respectively. A *p* value < 0.05 was considered statistically significant.

Qualitative analysis began with a holistic reading of the focus group transcript to capture the overall tone, discursive emphases, and initial impressions of the meanings attributed by participants. This approach facilitated familiarization with the textual corpus and the establishment of a preliminary interpretive framework [51]. ATLAS.ti software, version 25, was used to support the coding, retrieval, and organisation of the analytical material [52].

Subsequently, an axial coding process was applied, organizing hermeneutic units into analytical matrices that grouped emergent categories and subcategories. This procedure enabled the identification of relationships among codes, thematic patterns, and the construction of a conceptual structure aligned with the study objectives [50,53]. Categories were enriched with representative verbatim quotations extracted directly from the focus group, enhancing interpretive depth and fidelity to participants’ discourse [54]. This systematic and reflexive approach contributed to a comprehensive understanding of the emotional, physical, and social impact of the intervention, integrating qualitative findings with results from the quantitative phase.

## 3. Results

The study initially included 64 older adults. During the study period, six participants withdrew due to health problems (n = 2), relocation (n = 1), or personal reasons (n = 3), resulting in a final sample of 58 participants. Their mean age was 72.1 years (range: 60–83 years), and 52 participants (89.7%) were women. The descriptive and anthropometric characteristics of the final sample are presented in Table 1.

**Quantitative Phase**

**Figure 2:**
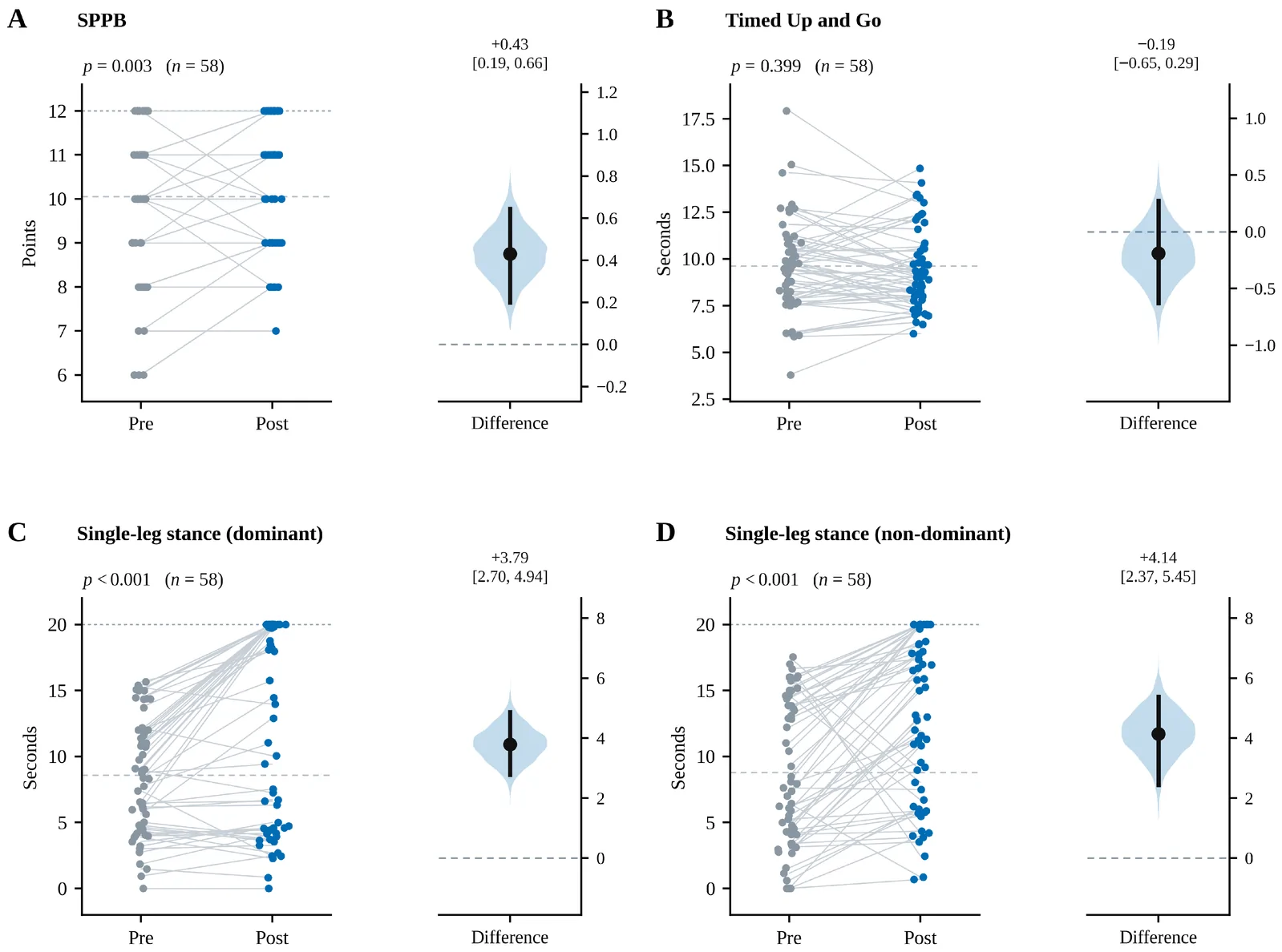
Pre− to post-intervention changes in physical function among participants who completed both assessments (n = 58). Changes are presented for (**A**) Short Physical Performance Battery score, (**B**) Timed Up and Go test, (**C**) dominant-limb single-leg stance, and (**D**) non-dominant-limb single-leg stance. Individual pre- and post-intervention observations are connected by lines. The estimation plots show the paired mean difference calculated as post-intervention minus pre-intervention values; the black point represents the mean difference, the vertical line represents its 95% confidence interval, and the shaded distribution represents the sampling distribution of the estimated difference.

SPPB scores increased significantly from 10.05 ± 1.76 to 10.48 ± 1.48 points (*p* = 0.003), whereas TUG performance remained unchanged (9.62 ± 2.43 vs. 9.43 ± 2.13 s; *p* = 0.399). Single-leg stance performance improved significantly in both the dominant limb (8.59 ± 4.53 vs. 12.38 ± 7.48 s; *p* < 0.001) and the non-dominant limb (8.77 ± 5.47 vs. 12.91 ± 6.21 s; *p* < 0.001) (Table 2 and Figure 2). Changes in physical function were mainly observed in SPPB performance and static balance, while TUG remained unchanged. Bilateral single-leg stance showed the largest effect sizes among the physical-function outcomes (Table 2 and Figure 2).

**Figure 3:**
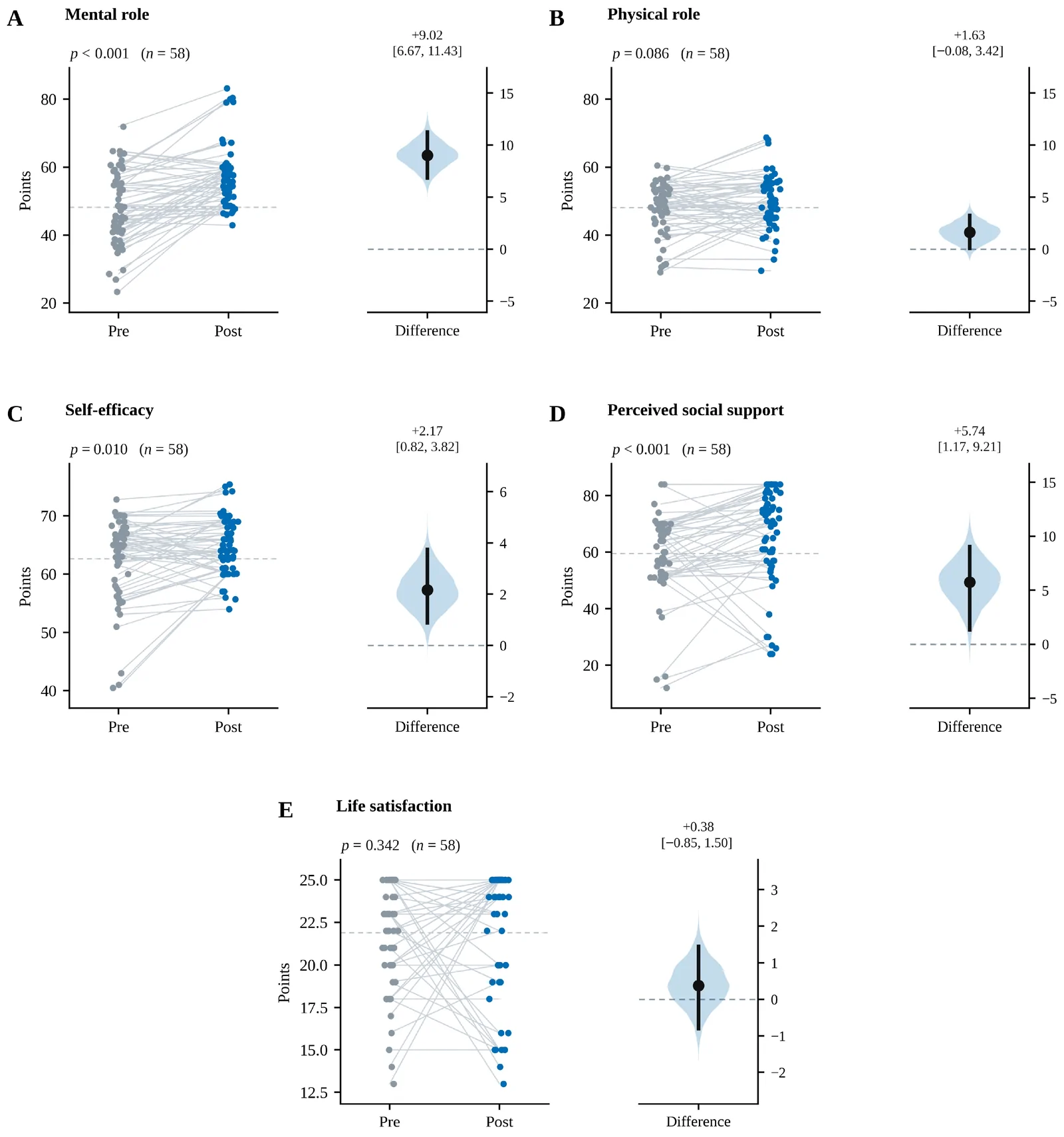
Pre −to post-intervention changes in psychological well-being and health-related quality of life among participants who completed both assessments (n = 58). Changes are presented for (**A**) SF-12 Mental Component, (**B**) SF-12 Physical Component, (**C**) general self-efficacy, (**D**) perceived social support, and (**E**) life satisfaction. Individual pre- and post-intervention observations are connected by lines. The estimation plots show the paired mean difference calculated as post-intervention minus pre-intervention values; the black point represents the mean difference, the vertical line represents its 95% confidence interval, and the shaded distribution represents the sampling distribution of the estimated difference.

Regarding psychological outcomes, the SF-12 Mental Component increased significantly from 48.17 ± 10.57 to 57.19 ± 9.06 points (*p* < 0.001), whereas the Physical Component remained unchanged (48.04 ± 7.40 vs. 49.66 ± 7.76 points; *p* = 0.086). Significant increases were also observed in general self-efficacy (62.65 ± 7.05 vs. 64.81 ± 4.91 points; *p* = 0.010) and perceived social support (59.48 ± 14.32 vs. 65.22 ± 17.19 points; *p* < 0.001). Life satisfaction did not change significantly (21.90 ± 3.08 vs. 22.28 ± 3.73 points; *p* = 0.342) (Table 2 and Figure 3). A similarly selective pattern was observed for the psychosocial outcomes, with changes concentrated in perceived mental health, self-efficacy, and social support, whereas perceived physical health and global life satisfaction remained stable. The SF-12 Mental Component showed the largest standardized change among the psychological outcomes (Table 2 and Figure 3).

**Qualitative Phase**

From the participants’ perspective, the folklore-based intervention with older adults was perceived as having a marked impact at the technical, emotional, and social levels. Focus group analysis revealed a positive appraisal of the cueca learning process, highlighting the development of specific bodily skills such as balance, coordination, and the execution of complex steps. The figure of the instructor emerged as a central reference, recognized for her affective pedagogy, patience, and capacity to adapt teaching strategies to the needs of the group. A total of 17 participants took part in this qualitative phase.

As shown in Table 3, participants also emphasized the emotional and relational dimensions of the learning process. At the emotional level, they reported overcoming barriers such as embarrassment and fear of ridicule, which facilitated the development of confidence, self-esteem, and personal security. At the social level, interpersonal bonds were strengthened through the formation of dance pairs and shared group interaction, consolidating folklore as a tool for integration, belonging, and community well-being.

Within the general experience of participation in folklore classes, three key categories emerged that structure older adults’ narratives. First, the figure of the instructor occupied a central role, characterized by pedagogical quality, patience, methodological adaptability, and personalized attention, resulting in high levels of appreciation from the group. Second, positive perceptions associated with the activity were identified, with emotions such as joy, enjoyment, and companionship being recurrent, reinforcing the playful and social dimension of learning. Finally, technical learning was recognized as a meaningful component of the experience, particularly in relation to the challenges involved in mastering cueca elements such as turns and footwork. These challenges were interpreted as valuable achievements and as a way of re-signifying this traditional dance through personal experience.

**Emotional and Social Impact**

Within the Emotional and Social Impact dimension, categories emerged that reflect marked transformations in participants’ psychological well-being and interpersonal relationships. As illustrated in Table 4, participation in folklore classes enabled older adults to overcome limiting emotions such as fear of ridicule and initial embarrassment, fostering more positive emotional states including joy, enthusiasm, and improved mood.

Participants’ narratives illustrate this emotional transition clearly: “I lost my embarrassment about looking ridiculous” and “we were worried before dancing, because of embarrassment and not knowing how to dance, but once we danced the fear went away.” These statements reflect a shift toward positive emotions, further reinforced by the festive and culturally meaningful context of the classes: “the music sounded very joyful, and it coincided with the national holiday celebrations.”

As shown in Table 4, the social connection category captures the strengthening of interpersonal bonds facilitated by shared dance practice and the relational structure of the sessions: “dancing connected us; we had our own dance partners.” The classes were perceived as spaces that encouraged interaction, conversation, and companionship, allowing participants to feel socially integrated.

**Satisfaction and Future Projections**

The Satisfaction and Future Projections dimension captures both the overall appraisal of the experience and participants’ expressed wishes regarding continuity and expansion of similar initiatives. As summarized in Table 5, older adults reported high levels of satisfaction with the workshop, highlighting enjoyment, meaningful learning, and the supportive instructional environment.

Within this dimension, participants’ statements clearly reflect the perceived value of the intervention: “we gave the cueca classes a ten,” grounding their evaluation in adaptive methodology and a respectful learning environment: “we enjoyed it, had a good time, and learned,” and “no one here was forced to attend the classes.”

The Methodological appraisal of the instructor highlights the pedagogical strategies implemented and their impact on learning outcomes, particularly the ability to adjust content to participants’ capabilities and to provide individualized support. Finally, the Projections and desire for continuity category captures participants’ interest in repeating the experience and extending similar initiatives in the future, including positive spillover effects within families: “it motivates me even more because my daughter enrolled in a folklore workshop.”

Several of these qualitative observations were consistent with the changes identified in the quantitative phase. Improvements in SPPB and bilateral single-leg stance paralleled participants’ reports of better balance, coordination, and greater mastery of complex dance movements. Changes in the SF-12 Mental Component, self-efficacy, and perceived social support were also reflected in accounts of reduced embarrassment and fear of ridicule, greater confidence, positive emotional experiences, and stronger interpersonal bonds. In contrast, TUG and life satisfaction did not change significantly, showing that the experiences reported by participants were not reflected equally across all quantitative outcomes. The qualitative findings therefore provide additional context for the physical, emotional, and social changes observed during the intervention.

## 4. Discussion

Overall, participation in the community-based theory-practice program grounded in Chilean folk dances was followed by significant improvements in SPPB performance, bilateral single-leg stance, the SF-12 Mental Component, self-efficacy, and perceived social support, whereas TUG, the SF-12 Physical Component, and life satisfaction did not change significantly. Complementarily, qualitative analysis of the focus group revealed that this experience was perceived as deeply enriching, fostering technical learning, the overcoming of emotional barriers, and the strengthening of social bonds. The instructor’s role, highly valued for her affective pedagogy and capacity for methodological adaptation, emerged as a key facilitator of the process. This convergence of findings suggests that interventions based on cultural practices may generate both measurable changes in selected health domains and subjective transformations that reinforce inclusion, motivation, and a sense of community.

The improvement observed in the total SPPB score reflects gains in components integrating balance, lower-limb strength, and gait speed [35]. This finding is consistent with recent studies employing community-based interventions and low-impact activities to enhance physical function in older adults [24,25,26,27]. Ji et al. [55] reported significant improvements in SPPB following a 12-week exercise and nutrition intervention in older Korean adults with functional sarcopenia, whereas Liang et al. [56] showed that 12 weeks of home-based strength and Tai-Chi exercise snacking increased SPPB scores in older adults in the United Kingdom. Together, these findings suggest that structured community-based programmes can improve lower-limb functional performance in later life.

Among the possible explanations for the observed improvements in physical function is the neuromuscular adaptation induced by the systematic practice of rhythmic, sequential, and coordinated movements characteristic of folk dance [57,58]. Sustained participation in this type of activity may have promoted greater neuromuscular efficiency, reflected in improved performance on tasks requiring the integration of balance, lower-limb strength, and mobility.

Beyond repeated muscular practice, dance may differ from non-musical exercise because movement is continuously organized by rhythmic auditory cues and changing spatial demands. Synchronizing steps to music may support temporal anticipation and auditory-motor coupling, while turns, head movements, changes in direction, weight transfers, and transitions between double- and single-leg support challenge the integration of visual, vestibular, and proprioceptive information required for postural control [19,59]. These processes provide a plausible interpretation of the improvements in single-leg stance, although sensory integration was not directly assessed in the present study [19,59,60].

Aging is associated with the progressive loss of fast motor units, which are partially reinnervated by slow motor neurons, resulting in larger but less efficient motor units [29]. This process compromises the ability to respond to rapid or complex motor demands, affecting essential functions such as gait and balance [29,61]. However, recent evidence suggests that functional or neuromuscular training programs, even at moderate intensity, can induce positive neurophysiological adaptations, including increased cortical activation and higher firing rates of remaining motor units [62].

Within this context, the intervention implemented in the present study based on dance sequences adapted to participants’ functional capacities may have stimulated these mechanisms, promoting more efficient and coordinated activation of muscle groups involved in balance and movement [63]. Although molecular or neurophysiological changes were not directly assessed, future research should incorporate biomarkers and neuroimaging techniques to confirm the activation of these mechanisms and to better understand the effects of motor–folklore training on the neuromuscular system of older adults.

In contrast, the TUG test did not show significant changes after the 9-week intervention. This test assesses a complex motor sequence involving standing up from a chair, walking three meters, turning, returning, and sitting down. Its execution requires not only general functional mobility but also lower-limb power, reaction speed, and executive motor control [64]. These physiological domains have been shown to be particularly sensitive to high-intensity or progressively overloaded training programs [65], conditions that were not central to the present protocol, which emphasized moderate and progressive loads tailored to participants’ baseline functional status. Moreover, the fact that baseline TUG values were within adequate functional ranges suggests the presence of a floor effect, since shorter TUG times denote better performance, which may have limited the detection of quantifiable improvements following the intervention.

Regarding psychological well-being, a significant increase was observed in the Mental Component of the SF-12, indicating improved perceived mental health following the intervention. This finding aligns with Sánchez-Alcalá et al. [66], who implemented a 12-week dance program in older adults with mild cognitive impairment and reported significant improvements in this component. The combination of rhythmic movement, cognitive engagement, social interaction, and culturally meaningful expression may have contributed to this change. Neurotrophic pathways, including brain-derived neurotrophic factor (BDNF), have been proposed as one possible biological explanation for responses to dance and aerobic exercise; however, BDNF and other biomarkers were not measured in this study, and this mechanism should therefore be regarded only as a hypothesis rather than an explanation demonstrated by the present data [20,67,68]. Recent studies have also emphasized the potential of dance programs to support emotional well-being and perceived vitality in older adults [21,69].

Nevertheless, it is important to consider that the benefits observed in the mental dimension of the SF-12 may not be attributable exclusively to the physical component of the intervention. Sessions included a theoretical–reflective space aimed at re-signifying movement from an educational, emotional, and cultural perspective. This component promoted the expression of personal experiences, collective dialogue regarding the value of folklore, and recognition of corporeality as an identity-related dimension. Such instances of encounter and reflection may have contributed to improved mental health perception by strengthening a sense of belonging, emotional validation, and the construction of shared meanings factors recognized as protective of psychological well-being in older age [5].

Life satisfaction did not change significantly following the intervention despite participants’ positive qualitative appraisal of the program. One possible explanation is the high baseline score (21.90 of a maximum of 25 points), which left limited room for measurable improvement and may have produced a ceiling effect. In addition, global life satisfaction may be less responsive to a 9-week intervention than more proximal outcomes such as perceived mental health, self-efficacy, or social support. Thus, the reported enjoyment, sense of belonging, and emotional benefits may represent meaningful experiences that were not fully captured by change in a global life-satisfaction measure. This interpretation remains compatible with evidence linking group dance participation with affective well-being and belonging [26,30,70].

Significant increases were observed in both self-efficacy and perceived social support. The improvement in self-efficacy may be attributed to repeated experiences of bodily mastery, which strengthen motivational mechanisms and perceived control over one’s own actions. Consistent with this, Hwang and Chung [71] argue that structured dance programs foster self-confidence by providing continuous opportunities for supervised motor success, thereby reinforcing beliefs in personal capacity to face physical and emotional challenges. The increase in perceived social support is also consistent with the qualitative reports of stronger interpersonal bonds, dance partnerships, and group connection. Although meaningful social support generally requires repeated and sustained interactions [72,73], the twice-weekly paired and collective activities may have provided sufficient opportunities for reciprocal contact and social reinforcement during the intervention.

Moreover, as previously noted, qualitative findings from the focus group revealed that the folklore intervention was experienced as deeply enriching, intertwining technical learning, emotional transformations, and meaningful social bonds. This experience aligns with Buckland [74], who argues that traditional dance not only transmits cultural knowledge but also activates bodily and affective memories that strengthen a sense of belonging and authenticity. In the present study, participants re-signified cueca as an accessible and valuable practice, overcoming initial barriers such as embarrassment and fear of ridicule, which translated into improved mood and personal confidence.

The emotional component of dance has been widely documented in the literature. Tleubayev et al. [75] highlight how folklore mobilizes spontaneous emotions and generates positive mood states, while Fong Yan et al. [76] demonstrate that dance-based interventions can influence psychological variables such as self-efficacy, life satisfaction, and the Mental Component of health-related quality of life. The present findings were partially consistent with this evidence: the Mental Component, self-efficacy, and perceived social support improved, whereas life satisfaction remained unchanged. Together with the qualitative findings, these results indicate that expressive movement may influence several, but not necessarily all, dimensions of subjective well-being.

From a social perspective, the focus group evidenced that dance facilitated interaction, companionship, and the formation of affective bonds, particularly through dance partnerships and group interaction. Cross and Tolbert [77] argue that dance, as a synchronized and collaborative practice, promotes social cohesion and a sense of community. This relational dimension was a salient feature of the programme, as participants described feeling integrated and recognized within the group.

Finally, the figure of the instructor emerged as a central element of the experience, valued for her affective pedagogy, patience, and capacity for methodological adaptation. Bannon and Sanderson [78] emphasize that the role of the dance instructor extends beyond technical instruction, acting as an emotional mediator and facilitator of meaningful learning. In this regard, Sööt and Viskus [79] underscore the importance of flexible, learner-centered pedagogical strategies, particularly in community contexts and with vulnerable populations. The instructor in the present study was recognized for her ability to personalize support, foster trust, and adapt content to group capabilities, which participants consistently identified as a facilitator of their engagement.

### Strengths and Limitations

This study presents several methodological strengths. First, the mixed-methods design constitutes a substantial strength, as it integrates quantitative and qualitative analyses, allowing exploration not only of objective intervention effects but also of participants’ perceptions and subjective experiences. This methodological integration supports a more comprehensive understanding of the phenomenon under study. Second, validated outcome measures were combined with session-level monitoring through perceived exertion and triaxial accelerometry, which supported safe, progressive pedagogical adjustments during the program; accelerometry was used for monitoring rather than as an outcome measure. However, several limitations should be considered when interpreting the findings. The single-group pre–post quasi-experimental quantitative design, without a control group, limits the ability to establish direct causal relationships between the intervention and observed changes. Additionally, non-probabilistic convenience sampling introduces potential selection bias and restricts the generalizability of findings to other older adult populations. The quantitative analyses were based on the 58 participants who completed both assessments, and the exclusion of six participants lost to follow-up may have introduced attrition bias. Participants who discontinued the programme were not assessed at follow-up, which precluded analyses incorporating their post-intervention data; future studies should assess all enrolled participants regardless of adherence. The marked predominance of women likely reflects the composition of the community-center sample reached through convenience recruitment; however, it limits external validity and precludes conclusions regarding potential sex-related differences in response to the program. Future studies should therefore consider controlled designs, larger and more sex-balanced samples, and follow-up assessments to determine whether the observed changes are maintained over time. Research conducted across different community settings would also help establish the reproducibility and generalizability of culturally adapted folk-dance interventions. In addition, comparing different intervention durations and levels of progression could help identify the characteristics of the program that are most closely associated with sustained participation and changes in physical and psychosocial outcomes.

## 5. Conclusions

The findings of this study indicate that participation in a motor education program grounded in Chilean folk dances was associated with improvements in specific domains of physical function and psychological well-being among older adults by integrating technical, emotional, and social components within an embodied learning experience. When pedagogically mediated, folk dance may support neuromuscular processes, strengthen self-efficacy, and enhance perceived social support while also re-signifying movement as a cultural and identity-based expression. The convergence of quantitative and qualitative findings supports the value of structured, culturally situated, and affectively meaningful interventions for older adults. From a practical perspective, this model could be implemented in community centers and similar settings as a group-based strategy that combines progressive motor practice, culturally meaningful content, and adaptable teaching methods. Its flexible structure may facilitate application in heterogeneous groups of older adults and adaptation to other cultural contexts, particularly in Latin America, where traditional dances constitute a shared cultural resource for community-based healthy ageing initiatives.

## Figures and Tables

**Table 1:** Descriptive characteristics of the final sample (n = 58).

	(Mean ± SD)
Age (years)	72.1 ± 5.75
Height (m)	1.54 ± 0.09
Body mass (kg)	69.3 ± 13.1
Body mass index (kg/m^2^)	29.3 ± 4.81
Waist circumference (cm)	96.9 ± 13.4

Values are presented as mean ± standard deviation; kg, kilograms; m, meters; cm, centimeters.

**Table 2:** Pre–Post Changes in Physical Function and Psychological Well-Being.

	Pre (Mean ± SD)	Post (Mean ± SD)	Mean Difference [95% CI]	Effect Size	*p* Value
SPPB (points)	10.05 ± 1.76	10.48 ± 1.48	+0.43 [0.19, 0.66]	0.27	0.003 **
TUG (s)	9.62 ± 2.43	9.43 ± 2.13	−0.19 [−0.65, 0.29]	−0.08	0.399
Single-leg stance—dominant (s)	8.59 ± 4.53	12.38 ± 7.48	+3.79 [2.70, 4.94]	0.61	<0.001 ***
Single-leg stance—non-dominant (s)	8.77 ± 5.47	12.91 ± 6.21	+4.14 [2.37, 5.45]	0.71	<0.001 ***
Physical Component (points)	48.04 ± 7.40	49.66 ± 7.76	+1.63 [−0.08, 3.42]	0.21	0.086
Mental Component (points)	48.17 ± 10.57	57.19 ± 9.06	+9.02 [6.67, 11.43]	0.92	<0.001 ***
Life satisfaction (points)	21.90 ± 3.08	22.28 ± 3.73	+0.38 [−0.85, 1.50]	0.11	0.342
Perceived social support (points)	59.48 ± 14.32	65.22 ± 17.19	+5.74 [1.17, 9.21]	0.36	<0.001 ***
Self-efficacy (points)	62.65 ± 7.05	64.81 ± 4.91	+2.17 [0.82, 3.82]	0.36	0.010 *

** Values are presented as mean ± standard deviation (SD); 95% confidence intervals (CI). SPPB = Short Physical Performance Battery; TUG = Timed Up and Go; s = seconds. * *p* < 0.05; ** *p* < 0.01; *** *p* < 0.001.

**Table 3:** Emergent category matrix within the General Experience dimension.

Dimension	Category	Emergent Codes	Description
General experience	The instructor	“Good instructor”, “excellent instructor”, “patience”, “varied”, “reinforced in a personalized way”, “pedagogue”	Highly positive appraisal of the instructor, emphasizing her patience, pedagogical quality, methodological adaptability, and individualized attention.
General experience	Positive perceptions	“Fun”, “I laughed”, “helped us socialize”, “I loved it”, “we put effort into it”	Positive emotions, enjoyment, and motivation associated with the classes, highlighting companionship and shared group experiences.
General experience	Technical learning	“Learning to dance cueca”, “technique”, “turns”, “footwork”, “watching huasos dance”, “watching cueca and understanding it”	A technically challenging yet rewarding learning process, particularly related to mastering cueca steps and movement patterns.

**Table 4:** Emergent category matrix within the Emotional and Social Impact dimension.

Dimension	Category	Emergent Codes	Description
Emotional and social impact	Emotional transformations	Overcoming embarrassment, confidence, loss of fear, joy, energy, mood	Folklore classes were perceived as a space that facilitated overcoming emotional barriers such as fear of ridicule and initial embarrassment, leading to positive emotional states and improved mood.
Emotional and social impact	Social connection	Peer bonding, dance pairs, group connection, interaction, companionship	Participation promoted meaningful social interactions, strengthening interpersonal bonds and fostering a sense of belonging and community.
Emotional and social impact	Instructor appraisal (social)	Affection toward the instructor, pedagogical recognition, patience, emotional closeness	The instructor was highly valued for creating an emotionally safe, supportive, and motivating environment that facilitated participation and emotional expression.

**Table 5:** Emergent category matrix within the Satisfaction and Future Projections dimension.

Dimension	Category	Emergent Codes	Description
Satisfaction and future projections	Positive evaluation of the experience	“We gave the cueca classes a ten”, “we had a good time”, “we enjoyed it”, “we learned”, “no one was forced”	Participants expressed high satisfaction with the workshop, emphasizing enjoyment, learning, a positive atmosphere, and voluntary participation.
Satisfaction and future projections	Methodological appraisal of the instructor	“The instructor modified the dance”, “reinforced in a personalized way”, “used different methodologies”, “adapted”	Strong appreciation of the instructor’s ability to adapt content, personalize instruction, and apply diverse pedagogical strategies to facilitate learning.
Satisfaction and future projections	Projections and desire for continuity	“That it be repeated with the same instructor”, “another workshop for us”, “my daughter enrolled in a folklore workshop”	Clear desire for continuity of the experience and evidence of broader positive impact beyond the group, motivating future participation and engagement.

## Data Availability

The data that support the findings of this study are available from the corresponding author upon reasonable request.

## Abbreviations

The following abbreviations are used in this manuscript:

BMI	Body mass index
EFAM-Chile	Functional Examination of Older Adults—Chile
HRmax	Age-predicted maximal heart rate
RPE	Rating of perceived exertion
SF-12	12-Item Short Form Health Survey
SPPB	Short Physical Performance Battery
TUG	Timed Up and Go
WHO	World Health Organization
